# Book Reviews

**Published:** 1833-04

**Authors:** 


### CRITICAL ANALYSES.

Quæ laudanda forent, et quæ culpanda, vicissim,
Illa prius, cretâ; mox hæc, carbone, *notamus*.—Persius.

*Researches on the Pathology and Treatment of some of the most important Diseases of Women.* By Robert Lee, M.D., F.R.S., Physician Accoucheur to the British Lying-in Hospital and the St. Mary-le-bone Infirmary; Lecturer on Midwifery in the School of Webb street.—8vo. pp. 220. Highley, London.

It is but within the last few years that any precise or fixed opinions have been formed respecting the pathology of two frequent and serious diseases of women, namely, "puerperal fever" and "phlegmasia dolens;" and our readers are doubtless aware that Dr. Lee has mainly contributed to our improvement, and that to his labours we are indebted for by far the most satisfactory evidence that has been brought forward of the real nature of these maladies. The work before us contains a connected view of the results of Dr. Lee's researches and labours upon these diseases: he has before published detached papers upon the same topics in the Medico-Chirurgical Transactions, but in this volume he gives us additional facts and additional arguments in support of his opinions.

Impressed with the necessity of rigidly investigating the pathology of puerperal fever, in order to reconcile the discordant statements of those who had previously written upon the subject, Dr. Lee determined to avail himself of every opportunity that occurred in his public and private practice, which could enable him to arrive at more definite and satisfactory conclusions than his predecessors had obtained. From the 1st of January, 1827, to the 1st of October, 1832, 172 cases of well-marked puerperal fever came under his immediate observation in private practice, and in the British Lying-in Hospital and other public institutions in the western districts of London. The symptoms and progress of these cases were watched with close attention; the effects of the different remedies employed were observed, and, where death took place, the alterations of structure in the uterine and other organs were carefully examined. Of fifty-six cases which proved fatal, the bodies of forty-five were examined, and in all were found some morbid change, decidedly the effect of inflammation, either in the peritoneal coat of the uterus or uterine appendages, in the muscular tissue, in the veins, or in the absorbents of the uterus; accounting very satisfactorily for the constitutional disturbance observed during life.

"The peritoneum and uterine appendages were found inflamed in thirty-two cases; in twenty-four, there was uterine phlebitis; in ten, there was inflammation and softening of the muscular tissue of the uterus; and in four, the absorbents were filled with pus. These observations are therefore subversive of the general opinion now prevalent, that there is a specific, essential, or idiopathic fever, which attacks puerperal women, and which may arise independent of any local affection in the uterine organs, and even prove fatal without leaving any perceptible change in the organization of their different textures. As the constitutional symptoms thus appear to derive their origin from a local cause, it would certainly be more philosophical, and more consistent with the principles of nosological arrangement, to banish entirely from medical nomenclature the terms puerperal and child-bed fever, and to substitute that of uterine inflammation, or inflammation of the uterus and its appendages in puerperal women. Puerperal peritonitis and peritoneal fever are terms not less objectionable than puerperal fever; for in many fatal cases there is no proof whatever of the existence of any morbid affection of the peritoneum." (P. 3.)

No experience of our own would justify us in demurring to these opinions, viewing them even in their fullest extent; but we must observe, that M. Tonnellé, whose very valuable series of papers on Puerperal Fevers were published in vol. 8, new series of this Journal, admits an anomalous or ataxic form of the disease, which he rarely observed except during the course of an epidemic. In this species of the disease, M. T. states that, "upon dissection, there was scarcely any appreciable alteration detected, and certainly none which would explain the death of the patient."* We are inclined to believe that such cases are extremely rare, but the authority of M. Tonnellé is too trustworthy to justify us in refusing to admit their occasional occurrence.

When we consult the works of the most celebrated writers on puerperal fever in this country, it clearly appears that they all describe the disease as commencing with a sense of soreness, or exquisite tenderness, in the region of the uterus; and that, where it proves fatal, the appearances on dissection afford unequivocal proofs of inflammation of one or more of the pelvic and abdominal viscera. The bodies of fifty-six women were examined who had died of puerperal fever in the general hospital at Vienna, in 1819; and in all of these, with the exception of two, traces of inflammation were found in one or more of the abdominal viscera.

After referring to various writers upon the subject, Dr. Lee mentions it as a curious circumstance, that in none of their works has the most remote allusion been made to inflammation of the veins, absorbents, or any of the other structures of the uterus, except the peritoneal covering; though several authors have accurately described the symptoms which characterize their morbid states. Dr. Lee enumerates the following as the principal varieties of inflammation of the uterus and its appendages in puerperal women.

"1. Inflammation of the peritoneal covering of the uterus and of the peritoneal sac.

"2. Inflammation of the uterine appendages: viz. the ovaria, Fallopian tubes, and broad ligaments.

"3. Inflammation of the mucous and muscular, or proper tissue of the uterus.

"4. Inflammation and suppuration of the absorbent vessels, and veins of the uterine organs." (P. 18.)

These varieties of uterine inflammation may take place independently of each other, though they are most frequently met with in combination.

Inflammation of the uterine organs, like inflammation of the lungs and other maladies which assume an epidemic form, occurs more frequently at one season than another; and at one period the peritoneum is the tissue most commonly affected, whilst, at other seasons, the deeper-seated tissues are almost invariably found affected by the inflammation.

* London Med. and Phys. Journal, October 1830, p. 309, where cases of this variety of puerperal fever are given.—Rev.

"That there is no essential difference between these varieties of uterine inflammation is proved by the circumstance, that in the course of a few days, in the same ward of the British Lying-in Hospital, and in patients who were placed in contiguous beds, during the prevalence of the epidemic, when the disease appeared to be communicated from person to person, peritoneal inflammation, uterine phlebitis, and the other varieties enumerated, all occurred in their most characteristic form. In some patients the local and constitutional symptoms indicated the presence of acute inflammation of the serous covering of the uterus; and in those cases where active depletion was employed at the commencement of the attack, most frequently a speedy recovery took place. In other examples, at the onset of the disease, there was comparatively little pain in the region of the uterus, the pulse was from the beginning rapid and feeble, and the symptoms were such as to contraindicate the use of bloodletting and cathartics. Such cases usually terminated fatally, in defiance of local bleeding and the exhibition of mercury and opium, and other remedies; and, on examination after death, either the veins, the muscular structure, or the appendages of the uterus, were found to be the textures most frequently inflamed." (P. 19.)

"*Inflammation of the Peritoneal Covering of the Uterus, and of the Peritoneal Sac.*" The symptoms are great tenderness of the hypogastrium increased by pressure, with pyrexia. When the attack is violent, the patient generally lies upon her back, with the knees drawn up to the trunk of the body. The abdomen is at first soft and flaccid, and, except in the region of the uterus, is frequently not affected by pressure. Though an enlarged and painful state of the uterus is never altogether wanting, "yet the pain often undergoes exacerbations similar to after-pains, and is frequently mistaken for these by careless observers," and the true character of the disease is overlooked until a great part of the peritoneal sac is inflamed.

"The whole abdomen then becomes swollen and tympanitic, and the pain either wholly subsides, or becomes still more intense than at the commencement. Diarrhœa and vomiting of black or dark-green-coloured fluids follow, as in other fatal inflammatory diseases of the abdominal viscera, the pulse becomes extremely rapid and feeble, the tongue dry and brown, the lips and teeth are covered with sordes, and death follows at no very remote period." (P. 21.)

In different individuals, the disease varies considerably in its commencement. Sometimes the attack of pain is sudden, at other times the ordinary increased sensibility of the uterus, remaining after natural labour, passes insensibly into the acute pain increased by pressure, the chief pathognomonic symptom of this affection. At the accession, there are generally rigors, which are partial and slight, or general, and so severe as to produce shivering of the whole body.

"The cold stage, after a longer or shorter duration, passes away, and is succeeded by heat of skin, suffusion of the countenance, acceleration of the pulse, and quick respiration, thirst, frequently nausea or vomiting, and vertigo or intense pain across the forehead. Cough is also a common symptom of the disease. The rigors precede, accompany, or follow the increased sensibility of the uterus. In some of the most severe cases there has been no distinct rigor; but a quick pulse, hot skin, and hurried respiration, have rapidly succeeded to the uterine pain. In most of the fatal cases the countenance has, from the commencement, been anxious and pallid, and the extremities cold.

"There is no uniformity observable in the appearance of the tongue in puerperal peritonitis. It is sometimes entirely covered with a thin, moist, white, or cream-like film; at other times, it is of a deep red, or brown colour in the centre, with a thick yellow or white fur on the edges.

"The lochia are often entirely suppressed; in other cases, only diminished in quantity. In some instances they have an offensive odour. The mammæ usually become flaccid; yet, in some fatal cases, the milk has been secreted until a short period before death. The urine is often passed with pain and difficulty." (P. 21.)

*Diagnosis.* Dr. Lee states that this variety of uterine inflammation is often confounded with disordered states of the intestinal canal: irregular spasmodic contractions of the uterus, which constitute after-pains; hysteralgia; and "simple suppression of the lochial discharge." If by "simple suppression of the lochial discharge," Dr. Lee would imply the mere cessation of the discharge, without its being accompanied by any other symptoms indicative of peritoneal inflammation, we hope and believe that no practitioner would commit the blunder that is here supposed.

In cases of disordered states of the bowels after delivery, the pain is from the commencement diffused over the whole abdomen; it is a griping rather than an acute pain, does not commence in the region of the uterus, and is but little, if at all, aggravated by pressure. The abdomen is generally soft, puffy, and distended; the tongue loaded; there is thirst and headach; neither the lochia nor the secretion of milk are suppressed. The febrile attack is usually preceded by symptoms of derangement of the bowels.

Puerperal peritonitis is generally developed before the end of the fourth day after delivery, and sometimes even within twenty-four hours; whereas, this affection rarely appears until the termination of the first week.

"It is sometimes difficult to distinguish inflammation of the peritoneum from after-pains and hysteralgia. Where the pulse is accelerated, the remissions of pain incomplete, the lochia scanty or suppressed, and the hypogastrium tender on pressure, we shall arrive at a correct diagnosis, by considering the peritoneal coat of the uterus in a state of congestion and inflammation, and employing antiphlogistic treatment. There are few puerperal women, except those of a feeble and irritable constitution, or who have been previously exhausted by profuse hemorrhage, or some chronic disease, who are seriously injured by cautious depletion, local or general; and where death has followed the abstraction of sixteen or twenty ounces of blood from the arm, the fatal result may fairly be attributed to disease, and to the neglect of the remedy rather than to its abuse. In cases of intestinal irritation, I have often found the local abstraction of blood followed by decided relief: and the same holds true with respect to the severe irregular pains without inflammation, which often occur subsequently to delivery, and do not yield to the ordinary means of treatment." (P. 23.)

Nine fatal cases, with the dissections, are detailed, to illustrate the phenomena of puerperal peritonitis and inflammation of the uterine appendages.

Dr. Lee next offers some remarks on *inflammation and softening of the proper or muscular tissue of the uterus.* In all the cases of this kind which he has observed, the resources of nature and of art have proved equally unavailing in arresting its fatal course. In most of his observations upon this subject, Dr. Lee coincides with M. Tonnellé,* whose opinions he quotes. Upon one point they differ. Dr. L. says, "that the destruction of the healthy organization of the proper and internal tissues of the uterus is the consequence of an inflammatory process, and not of any peculiar specific action of the parts, or an altered state of the blood, as some German and French pathologists have maintained, may safely be inferred from the symptoms," &c. M. Tonnellé,† on the other hand, remarks, that in ramollissement of the uterus, the constant and determinate symptoms of inflammation are wanting; that inflammation does not destroy life with such terrible rapidity, especially when it attacks an organ which is not immediately necessary to the existence of the individual; and that it appears evident, therefore, that inflammation in such cases is but an accessory phenomenon, and a sort of veil behind which is concealed the really active cause. He further states, that he has many reasons for believing that this disease of the uterus depends upon a vitiated condition of the blood; but he confesses "that facts are yet wanting to confirm this, at present, hypothetical opinion."

Dr. Lee relates three cases of softening of the tissue of the uterus, and for additional examples of this formidable disease we refer to our Journal for October 1830.

"*Inflammation and Suppuration of the Absorbent Vessels of the Uterus.*" We believe that M. Dance was the first pathologist who observed this species of disease. M. Tonnellé has also recorded several examples of it;‡ but, although he was not aware of the fact, the following case had been published in this country

* London Med. and Phys. Journal. p. 300, for October 1830.—Rev.

† Ibid., p. 303.—Rev.

‡ Ibid., July 1830, p. 10.—Rev.

several months before he wrote upon the subject in the Archives Génèrales.

A woman, æt. thirty, in an advanced state of pregnancy, was admitted into that hospital on July the 1st, under the care of Mr. Cæsar Hawkins, in consequence of sloughing of the skin covering a diseased bursa of the patella. The removal of the bursa was followed by great constitutional disturbance, and, on the 14th, labour came on. Two days after, symptoms of uterine inflammation made their appearance, and on the eighteenth day death took place. Though the pain was relieved by bleeding, she never rallied after the attack. On examining the body, some puriform lymph was found in the pelvis, but there was no increase of vascularity in the peritoneum. In the broad ligaments some fluid was also effused, and on each side numerous large absorbent vessels were observed passing up with the spermatic vessels, to the receptacle of the chyle, which was unusually distended. All these vessels, and the reservoir itself, were filled with pus; but that in the receptacle was mixed with lymph so as to be more solid; the vessels themselves were firmer and thicker than usual. The thoracic duct was quite healthy. The uterus was scarcely contracted, and the internal surface of the lower half was soft and shreddy, and in a state of slough. The upper part, where no pus was found externally, was also healthy, or nearly so, on its inner surface.*

It will be seen, by referring to the essays of M. Tonnellé, already quoted, that inflammation of the absorbents of the uterus, of the receptaculum chyli, and thoracic duct, occurs frequently in puerperal women, and that it gives rise to the same constitutional disturbance as uterine phlebitis. It appears, indeed, as Dr. Lee observes, that these varieties of uterine inflammation are frequently combined; and it is probable that in both the purulent fluid is conveyed by the absorbents and veins into the mass of circulating blood. "The local symptoms of this affection are often so obscure as to escape detection during life; while the constitutional symptoms, which sometimes resemble in a striking manner the effects produced by specific poisons, are so virulent as not to yield to any remedies, however early and vigorously employed."

"*Inflammation of the Veins of the Uterus, or Uterine Phlebitis.*" In healthy women, uterine phlebitis sometimes commences within twenty-four hours after an easy labour, with pain more or less acute in the region of the uterus, accompanied or followed by a severe rigor, or rigors, suppression of the milk and lochia, acceleration of the pulse, cephalalgia, or slight incoherence, great general uneasiness, sometimes nausea, vomiting, and diarrhœa.

"These symptoms, after a short duration, are succeeded by increased heat, tremors of the muscles of the face and extremities, rapid feeble pulse, anxious and hurried respiration, great thirst, with brown dry tongue, and frequent vomiting of green-coloured

* Med.-Chir. Trans. vol. xv. p. 64.

matters. The sensorial functions usually become much affected, and there is a state of drowsy insensibility, or violent delirium and agitation, which is soon followed by symptoms of extreme exhaustion. The whole surface of the body not unfrequently assumes a deep and peculiar sallow or yellow colour, or a petechial or vesicular eruption appears on different parts of the body. The abdomen also sometimes becomes swollen and tympanitic, and some of the remote organs of the body, such as the lungs, heart, brain, liver and spleen, or the articulations and cellular membrane, and muscles of the extremities, suffer disorganization, from a rapid and destructive congestion and inflammation.

"There is scarcely an organ which has not been observed to become secondarily affected from inflammation and suppuration of the uterine veins. The vessels of the brain sometimes become greatly congested, and lymph is effused upon the surface of the pia mater, or serum into the ventricles; portions of the cerebral pulp have become softened and disorganized, or purulent infiltrations have taken place into the cerebral substance.

"In other individuals, whose lungs had previously been healthy, a rapid and destructive inflammation of the pleura has taken place, or portions of the pulmonary texture have become condensed, of a dark red colour, or infiltrated with pus. In four cases, which have fallen under my observation, where there had been only obscure pain during life, with slight cough and dyspnœa, a copious effusion of lymph and serum was found within the cavities of the thorax; the pleura was covered with false membranes, and portions of the lung had fallen into a state of complete gangrene. In one individual the pleura had given way by sloughing, and the right side of the chest was found distended with air. Gangrene also sometimes takes place rapidly in those parts of the body on which the patient rests, and the same process is established in other soft parts where no pressure has been made. In a case related by Cruveilhier, which did not prove fatal, the nose became black and gangrenous." (P. 48.)

Amongst other occasional symptoms of uterine phlebitis, Dr. Lee mentions a sudden and destructive inflammation of the eyes, from which patients have been deprived of sight many days before the termination of life. In two cases which came under his care, the conjunctiva of both eyes, without much pain, suddenly became intensely red, the cornea opaque, and the eyelids much swollen, and under their lining membrane a large serous deposition took place; lymph and pus were also effused into the anterior chamber, and in one the cornea ultimately burst. Deposits or infiltrations of pus, of enormous extent, also took place into the cellular membrane, near the large joints, and between the muscles of the extremities. Upon this subject Dr. Lee quotes M. Tonnellé, to whose cases we refer.*

* London Med. and Phys. Journal, September 1830, p. 199 et seq.—Rev.

Sometimes uterine phlebitis begins at a later period after delivery, and in a much more obscure and insidious form, without pain or sense of uneasiness in the region of the uterus, or any other local symptom by which the affection can be recognized. The uterus may return to the reduced volume it usually assumes after delivery; the lochia may continue; and the inflammation and suppuration of the veins, which have caused the whole of the constitutional disturbance and destructive lesions in distant parts of the body, may have been wholly overlooked.

"Inflammation of veins rarely takes place in any part of the body, where it cannot be referred to a wound, or to some specific cause externally applied to the coats of the vessels. In uterine phlebitis, the inflammation cannot, it is true, be traced in all cases to the semilunar-shaped orifices in the lining membrane of the uterus which communicate with the sinuses, where the placenta has adhered; yet it scarcely admits of a doubt, that the frequent occurrence of the disease arises from the orifices of these veins in the lining membrane of the uterus, being left open after the separation of the placenta, by which a direct communication is established between the cavities of these veins and the atmospheric air, in a manner somewhat analogous to what takes place in amputation and other extensive wounds. Such a condition of the uterine veins, in consequence of the separation of the placenta, must be favorable to the production of inflammation; and inflammation once excited is seldom limited to these veins, but extends with greater or less rapidity along the continuous membrane of the uterine veins, to the spermatic or hypogastric, and from thence to the vena cava and its principal branches, which return the blood from the lower extremities."* (P. 51.)

The various alterations of structure produced by inflammation in the veins of the uterus, are accurately described by Dr. Lee. The vena cava itself does not always escape, the inflammation spreading to it from the iliac or from the spermatic veins.

"Uterine phlebitis appears to result from the mechanical injury inflicted upon the uterus, by protracted labour from the force required for the extraction of the placenta, in uterine hemorrhage, from retained portions of the placenta undergoing decomposition in the uterus, the application of cold, and perhaps of contagion, or from any of the causes which produce the other varieties of uterine inflammation. M. Dance considers deranged states of the lochia to be a frequent cause of the disease, but these are consequences, and not causes of uterine phlebitis." (P. 54.)

The origin and course of the symptoms above described are sometimes modified; but for the account of these variations we must refer to the work itself.

* See a paper by the author, in the Philosophical Transactions for 1832, on the Structure of the Human Placenta and its Connexion with the Uterus.

Uterine phlebitis is always dangerous, but not always fatal. That it often occurs in puerperal women, where it is not suspected during life, and where the symptoms are referred to other causes, Dr. Lee conceives to be demonstrated by the fact, that, in the spermatic and hypogastric veins of females advanced in years, calcareous concretions, and various other proofs of disorganization, have frequently been observed, which must have been produced by attacks of acute inflammation at some remote period. In many cases where the existence of uterine phlebitis was proved by the extension of the disease to the iliac and femoral veins, complete recovery followed.

The "historical view" of uterine phlebitis, given by Dr. L., is highly curious and interesting: it shows how near some pathologists have previously been to arrive at the convincing knowledge of its nature which we now possess, and for which we are so greatly indebted to his indefatigable industry and talent.

Twenty cases and dissections of examples of this disease are related in the work.

The causes of inflammation in the uterine organs of puerperal women are involved in great obscurity. Sometimes the inflammation is distinctly referrible to injury of the uterus during severe labour, to exposure to cold and moisture, or irregularity of diet soon after delivery: but frequently it arises in its most malignant form where none of these causes have been applied, and where it can only be referred to some peculiar noxious constitution of the atmosphere, or to contagion. Dr. Lee refers to many authorities who have touched upon the question of the contagious nature of the disease. As usual, upon such questions we have much diversity of opinion. For himself, Dr. L. says, that the facts he has observed, though they have led him to adopt the opinion that the disease is sometimes communicable by contagion, yet they have not been sufficiently numerous, or of so decisive a character, as to dispel every doubt on the subject. He states that it has occurred in many cases, in the most destructive form, where contagion could not possibly be supposed to have operated as the cause. In other instances, however, he adduces quite sufficient evidence to show the contagious nature of the disease. For example: in the last two weeks of September 1827, five fatal cases of uterine inflammation came under his observation. All the individuals so attacked had been attended in labour by the same midwife, and no example of a febrile or inflammatory disease of a serious nature occurred during that period among the other patients of the Westminster General Dispensary, who had been attended by the other midwives belonging to the institution. Other facts of a similar nature are mentioned, and Dr. L. concludes by remarking, that we certainly owe it as a duty to our patients to act as if the contagion always existed.

Upon all subjects connected with female diseases Professor Jörg's opinion is deserving of attention; and, upon reference to his work on the Diseases of Women, we find he is decidedly of opinion that "puerperal fever" is a contagious disease.* But whatever conclusion we may arrive at as to the contagious or non-contagious character of the disease usually called puerperal fever, the symptoms, morbid appearances, and influence of remedies, all prove, whatever the nature of the remote cause may be, that it acts by exciting inflammation of the uterine organs. It is, perhaps, difficult to determine whether this inflammation be of a common or specific kind. Dr. Lee mentions some facts which seem to prove that there is some connexion between erysipelas and puerperal fever.

*Treatment of Uterine Inflammation in Puerperal Women.* Sometimes the inflammatory disposition is so slight as to yield readily to opiates and fomentations. CHAUSSIER and others have been so convinced of the advantages of employing these remedies, with the view of preventing attacks of the disease, that they have caused all patients recently delivered to take at intervals Dover's powder, and to apply cataplasms. In cases of intestinal irritation, after-pains, and various spasmodic affections of the uterus and abdominal viscera, this plan of treatment will prove successful. In the milder forms of uterine inflammation, a spontaneous termination of the disease not unfrequently takes place: but when the inflammation is fully developed, and where it exists in a severe sporadic or epidemic form, local and general bleeding, and other antiphlogistic remedies, must be early and vigorously employed.

"In the treatment of puerperal fevers, the following are the principal objects we should keep in view: first, to subdue the local inflammation of the uterine organs; and secondly, to moderate the constitutional disturbance which the local inflammation invariably produces. In fulfilling these indications, no exclusive plan of treatment should be adopted; but we ought, according to the peculiarities of each case and stage of the disease, to employ blood-letting, mercury, opium, cathartics, diaphoretics, blisters, and whatever other means we can discover to possess any influence in controlling the disease." (P. 102.)

Dr. Lee highly appreciates the good effects of bleeding, but the results of his experience do not confirm the accuracy of the conclusions drawn by some authors, that in all cases the early employment of bleeding will cure the disease. "It is always an affection attended with great danger, and it not unfrequently runs its course rapidly to a fatal termination, in spite of the most prompt application of remedies."

In combination with bleeding, eight or ten grains of calomel, with antimonial powder and opium, should be given every three or four hours till the symptoms begin to subside. "Upwards of fifty

* "Die Ansteckung allein reicht hin, um das Uebel (Kindbettfieber) enstehen zu lassen, und die beste und dauerhafteste Gesundheit ist in diesem falle nicht im stande, gegen dasselbe zu schützen." *Handbuch der Krankheiten des Weibes.* Von Dr. J. C. G. JÖRG. Leipzig, 1821, p. 794.—REV.

grains of calomel have been given in many cases with decided benefit, and in two only out of 170 cases has the mouth been severely affected." After the second dose of calomel, Dr. Lee has often given, with advantage, a strong purgative enema, followed by a cathartic draught. In some cases, after the operation of the medicine, the pain of the uterus, which had only been relieved, completely subsided. Sometimes a second bleeding is necessary; but "however much the patient may complain of uterine pain, if the pulse exceed 120 and is feeble, and if the powers of the constitution be much reduced by the previous treatment, blood should not be taken a second time from the arm." If the pain continue undiminished six or eight hours after the first bleeding, or even later, and the pulse be full and not very rapid, and the strength of the patient but little impaired, a second bleeding of twelve or fourteen ounces may be practised.

"It ought, however, to be remembered, that much greater caution is required in having recourse to the second than the first bleeding in puerperal peritonitis; and where we are not convinced that it is absolutely necessary again to abstract blood from the arm, it is better to repeat the leeching. In no case of peritonitis which has fallen under my care has it appeared necessary or safe to bleed from the arm a third time, and in a very large proportion of cases only one bleeding has been had recourse to." (P. 104.)

Dr. Lee speaks very doubtfully of the effects of spirits of turpentine given internally. Doulcet's treatment by emetics, of the success of which such exaggerated reports were raised, is altogether condemned. "In no case have I considered it safe to administer emetics in any stage of the complaint, and I cannot conceive it possible for a case to occur in which the treatment should chiefly or exclusively be conducted on the plan of Doulcet."

Blisters, and the external application of oil of turpentine, often relieve the pain.

The use of general and local warm baths will depend upon the state of the patient. Recolin, Dance, and Tonnellé* highly recommend the injection of warm water into the vagina and cavity of the uterus. Dr. Lee has tried it several times with decided advantage.

During the first stage of the disease, stimulating remedies are of course improper; but when the inflammatory symptoms have been subdued, and the patient is in a state of great exhaustion, quinine, ammonia, wine, &c. sometimes produce the happiest effects.

"I cannot too strongly urge the necessity of continuing to employ these remedies whilst the slightest hope of recovery is entertained. I have seen several patients restored to health, where the pulse had risen to 160, and was so feeble as scarcely to be felt at the wrist; where there was constant delirium, and the most alarming prostration of strength. Recovery has even taken place, in some

* London Med. and Phys. Journal, December 1830, p. 509.—Rev.

cases which I have observed, where the abdomen has become tympanitic, and effusion to a considerable extent had taken place into the abdominal cavity. In no acute disease is it of greater consequence than in this now under consideration, that the patient should be visited by the medical attendant at short intervals, and that the effects of the remedies he prescribes should be narrowly watched." (P. 111.)

With regard to the treatment of inflammation of the uterine appendages, and of the deeper-seated tissues of the uterus itself, whether of the absorbents, veins, or of the muscular structure, the symptoms are, says Dr. L., from the commencement such as commonly contra-indicate the use of general bloodletting. If the local pain is severe, leeches and warm fomentations are the most appropriate remedies; "but, as far as my own observations extend, we are not at present in possession of any remedial means which effectually control those varieties of inflammation of the deeper-seated structures of the uterus which I have endeavoured to describe." The French physicians, however, believe that we possess a powerful remedy, even in the worst cases, in mercury, employed so as to excite salivation.*

*Prophylactic Treatment.* It is evidently of great importance to prevent, if possible, the occurrence of a disease so destructive and so unmanageable. For several days after labour, every puerperal woman should take as much care of herself as an individual who is recovering from an attack of continued fever, or inflammation of some important viscus. Fatigue, exposure to cold, and irregularity of diet, are carefully to be avoided. Acrid cathartics should not be given soon after delivery, and no unnecessary pressure should be made upon the abdomen. All manual operations are to be carefully and tenderly performed, and portions of placenta should be prevented from remaining to become decomposed within the uterus.

"I cannot conclude this important subject without pointing out the urgent necessity which there exists for a full investigation of the means best calculated to prevent the occurrence of puerperal fever or uterine inflammation in Lying-in Hospitals, where its dreadful fatality has been recorded by all writers since the foundation of these institutions. From the registers of the British Lying-in Hospital, the Maternité at Paris, the Dublin Lying-in Hospital, and the tables of M. De Châteauneuf, it is proved that the average rate of mortality greatly exceeds that of institutions where individuals are attended at their own habitations; and if it should ultimately appear that all precautions are unavailing in diminishing the numbers attacked by the disease, it becomes a subject deserving of the most serious consideration, on the ground of humanity, whether Lying-in Hospitals should not be altogether abolished, as injurious rather than beneficial to society. From what has fallen

* Ibid. p. 502.—Rev.

under my own observation in the British Lying-in Hospital, and other similar institutions in this metropolis, where the utmost attention is paid to ventilation and cleanliness, and where the wards are not over-crowded with patients, I cannot hesitate to express my decided conviction that, by no means hitherto discovered, can the frequent and fatal recurrence of the disease be prevented in Lying-in Hospitals, and that the loss of human life thereby occasioned completely defeats the objects of their benevolent founders." (P. 114.)

In the succeeding pages, Dr. Lee treats of crural phlebitis, which term he proposes in place of *phlegmasia dolens,* as more indicative of the pathological nature of the disease; many cases and dissections are given tending to establish the views he maintains upon this subject, and to which we have before referred.* Some examples of "crural phlebitis" in men are also related. The valuable and original paper contained in the first chapter of the second part of the work, "on the Connexion of the Placenta and Fœtal membranes with the Uterus" we published from the Philosophical Transactions:† in the present volume, however, it is somewhat enlarged. We shall probably have occasion again to refer to this subject in consequence of the very unjustifiable and unfounded charge of plagiarism which was recently brought forward against Dr. Lee, at the Westminster Medical Society.

The volume terminates with some brief remarks on *uterine hemorrhage.* This subject, however, is very briefly treated, and we therefore forbear from entering into a formal criticism of some practical opinions with which we cannot altogether coincide.

We cannot too strongly recommend Dr. Lee's work to the attention of the profession, and especially those who are particularly occupied in the management of female complaints. It contains by far the most valuable contribution to our knowledge, that has been published in this country upon the very important diseases on which it treats, and we may suggest that the study will be made additionally interesting by comparing the practical experience of Dr. Lee with that of M. Tonnellé, to whose valuable papers we have so often referred in the course of the present review. For the convenience of our readers, many of whom may probably not have the Archives Génerales in their possession, we have quoted the translation of M. Tonnellé's essays instead of the original; and we, therefore, take this opportunity of vouching for the fidelity with which they were transferred from the French journal to our own pages.

* London Med. and Phys. Journal, March 1830, p. 267.—Rev.
† Ibid. July 1832, p. 71.—Rev.

*An Introduction to the Atomic Theory; comprising a Sketch of the Opinions entertained by the most distinguished ancient and modern Philosophers with respect to the Constitution of Matter.* By Charles Daubeny, M.D. F.R.S., Professor of Chemistry in the University of Oxford.—8vo. pp. 147. Murray, London.

The theory of definite proportions, as taught by Dalton, and as now received universally throughout the whole of the philosophic world, is, perhaps one, of the most brilliant and important truths ever revealed and confirmed by experimental researches; being to chemistry what the laws of gravity are to astronomical science.

We need not remind our readers, that the author of this theory received, during the meeting of naturalists, held at Oxford last summer, one of the highest honours, and one of the most gratifying rewards which that far-famed university could bestow; being made, in public convocation, an honorary Doctor; along with three other celebrated philosophers, Brown, Faraday, and Brewster.

The work now before us is another welcome tribute of respect paid by the same university to the genius of Dalton; and we think that Dr. Daubeny has done well, not only by giving to the world a concise and familiar explication of Dalton's system, but also by having added the weight which his official situation as Professor of Chemistry in the university of Oxford must give to any doctrines he supports.

We are much pleased with some casual observations that occur in the preface, upon the attention that should be paid, and the encouragement afforded, to the study of the natural sciences: it shows that, although our universities are old, and the ancient regime is at times too long and too strictly adhered to, still the importance of modern sciences and modern discoveries are not wholly overlooked; and doubtless, by the praiseworthy exertions of their ardent admirers, rewards will be secured to, and honours regularly conferred upon, the students in these, as well as in the more ancient departments of philosophy.

Dr. Daubeny has divided his essay into four parts, or chapters, to which an appendix and some additional notes are affixed. In the first chapter, he gives a brief "sketch of the opinions entertained respecting the constitution of matter, before the laws of definite proportions were discovered." In the second "an outline of those modern discoveries respecting the definite proportions in which atoms combine, which seem to prove the existence of ultimate atoms." The third chapter is "on the applications of which the laws of definite proportions are susceptible;" and the fourth contains "arguments derived from other branches of modern science in favor of the existence of indivisible particles, and an inquiry as to how far the doctrine of definite proportions may have been anticipated by the ancients."

It will be seen from this prodromus, that, although the work is small, its scheme is comprehensive, and the field which the author passes over one that is fertile, not only with physical truths, but also with classical recollections; and these his previous academical pursuits would not allow the writer to neglect. It is, therefore, apparent that the volume is designed not so much for the mere chemical tyro, as for the scholar and the gentleman.

We shall proceed to make some few extracts from the several chapters, as samples of both matter and manner; and the following is a proof of the fair and candid manner in which the opinions of the ancients, by which some would swear, are discussed.

"Two opinions have divided the ancient as well as the modern world; the first, that matter is composed of an assemblage of particles incapable of farther division; the second, that there is no limit to its divisibility, the smallest conceivable body still consisting of an infinity of parts.

"For an exposition of the former doctrine we commonly appeal to the writings of the Epicureans; but the notion itself may he traced much farther back. It formed indeed the groundwork of the cosmogony of Democritus, and was by him derived from Leucippus, who is generally regarded as its author.

"It is however stated, that the same opinion was held by Moschus, a Phœnician, who is supposed by some to have flourished before the Trojan war, and if, as has been imagined, the monads of Pythagoras were corpuscular atoms, the Egyptians, from whom that philosopher derived so many of his tenets, may probably have a claim also to this.

"It has been likewise shewn by Mr. Colebrook that the Hindoos from a very early period have embraced the doctrine of atoms, although the actual date of the system of philosophy into which this opinion enters is not fully made out.

"According to Kanadi, the author whom he quotes, atoms constitute the last term of the division to which matter can be subjected. They are too small to be objects of sensation, for the particles of dust that are seen in a sunbeam, which are the most minute of visible things, are composed of several of them. They are simple and not compounded, otherwise the series would be endless, and were it pursued indefinitely, there would then be no difference of magnitude between a mustard seed and a mountain, a gnat and an elephant, each alike containing an infinity of particles. The ultimate atom therefore is simple. The first compound consists of two atoms, and the next of three double atoms; for if only two were conjoined, magnitude could hardly ensue from their union, since the latter must be produced either by the size or the number of the particles: it cannot be by their size, and must therefore be by their number. The atom, then, is laid down to be the sixth part of the mote which we see in the sunbeam.

"Such was the doctrine that appears to have been most current among the Hindoo philosophers; but Mr. Colebrook informs us, in another memoir, that it was objected to by the orthodox, some of whom, who professed to found their opinions on the text of the

Indian scriptures, even argued against the existence of a material world, as was the case with some of those who rejected the atomic theory in Greece, with Berkeley in England, and with the more modern school of natural philosophy hardly yet extinct in Germany.

"Nor is it surprising that notions which have stood their ground till the present advanced state of science, should have been broached at so early a period: as the first poets are pregnant with the grandest conceptions, so the earliest philosophers often light upon the most sublime truths; astonishing us with an intermixture of the noblest views of nature with the most crude and vulgar conceits, and often leaving to their successors little more than the task of selecting from the mass of error the grains of truth which are disguised by and confounded with it.

"Thus, in the writings of Lucretius, we are struck in one page by the philosophical spirit which seems to anticipate the discoveries of modern times, in propounding a system not very different from the doctrine of *latent heat*, and maintaining, in opposition to Democritus, that the descent of heavy and light bodies *in vacuo* is equally rapid; and in the next are provoked at the puerile manner in which the poet attempts to account for the independence of the Will, by imagining an occasional deviation from a straight line to take place spontaneously in some of his atoms, whilst descending through space.

"It is the same with that part of his system which relates to the formation of the material world; we shall see reason perhaps to consider the position, that all bodies are composed of a certain number of ultimate particles, more consistent than any other with the discoveries of the present day; but we are not therefore the less sensible of the absurdity of supposing the beautiful variety of nature to be the result of a fortuitous concourse of insentient atoms, differing from each other solely in the mechanical properties of size and figure.

"The doctrine itself is not the less probable, because it fails to account for every thing which some of its supporters pretended to deduce from it, neither has it any natural tendency to atheism, although adopted by a sect of philosophers, who fancied they could dispense in their systems with the intervention of a Deity.

"Nor do the original atomic theories appear to have been atheistical; on the contrary, the same philosophers who proposed this view of the subject considered matter, we are told, as wholly passive, and therefore admitted, as a necessary consequence, the existence of a moving principle which should be distinct from matter." (P. 4.)

We pass by the observations on the philosophy of Epicurus, because the extracts are made from Dr. Good's edition of Lucretius, which has already rendered the subject familiar, and the speculations of Boscovich, Leibnitz, Des Cartes, and Kant would require more space than we think they would deserve in our eclectic pages.

Mr. Higgins, of Pembroke College, seems to have had some notion of the prevalence of the atomic constitution of bodies; for, "in a work published by him in 1789, entitled 'A Comparative View of the Phlogistic and Anti-phlogistic Theories,' he distinctly states, that one ultimate particle of sulphur and one of oxygen constitute sulphurous acid, whilst one ultimate particle of sulphur and two of oxygen constitute sulphuric acid; and moreover that in the compounds of azote and oxygen the ingredients are to each other in the proportion of 1 to 1, 2, 3, 4, 5, respectively."

But even these views, crude as they were, forestalled the knowledge of the times; for chemical analysis had not then sufficiently advanced to allow his opinions to be either refuted or confirmed: nor was it until Dalton, in 1808, published the first volume of his "New System of Chemical Philosophy," that the subject assumed a determined form. He had, indeed, previously inculcated his principles in public lectures; and, in 1803, had communicated an outline of his speculations to the Manchester Society: still these were but faint shadows of his work.

With regard to the curious subject of *Isomorphous* and *isomeric* bodies, there are some very sensible though brief remarks.

"Professor Mitscherlich has rendered it probable, that several of the bodies belonging to the same group assume crystalline forms, which, if not absolutely identical, are at least nearly related one to the other; and from this it will follow, that, supposing such bodies to be severally united with an equal number of atoms of the same body, their figure ought still to correspond. Thus if we suppose sodium, potassium, calcium, iron, and manganese, to agree in the shape of their ultimate particles, they ought, if combined with equal proportions of oxygen, to assume nearly the same crystalline arrangement. Under such a supposition, therefore, they would be called, in Professor Mitscherlich's phrase, *isomorphous* bodies.

"The same remark applies to the electro-negative division in an equal degree; thus the hyposulphurous acid, and the protoxide of azote, may be isomorphous bodies, although whether they are in fact so ought not to be taken for granted, without positive proof. The same may be the case with the sulphurous, the carbonic, the selenious, and the antimonious, acids, and so with regard to all the other groups.

"Now it might be expected, that if any two electro-negative bodies, themselves isomorphous, and associated with an equal number of atoms of the same body, as of oxygen, be made to unite with the same base, the crystalline form of the resulting compounds should be related: for, the number and form of the atoms of which they are respectively made up being analogous, it is probable that the aggregate arising out of them should be so likewise.

"Thus, if lime and protoxide of iron are isomorphous, the carbonates of these bases ought to possess a similar crystalline form, and this Mitscherlich has shown to be the case.

"The same observation will apply to bodies belonging to the electro-positive class, when combined with the same acid; and the German chemist cites in his first memoir several examples of this kind, taken from the combinations of different bases with the sulphuric acid." (P. 73.)

And again:

" It is curious, that the crystalline form of arragonite corresponds as nearly to that of carbonate of lead and carbonate of strontian, as that of common carbonate of lime does to carbonate of iron; so that we may perhaps conclude, that lime is capable (like sulphur) of assuming two crystalline arrangements, the one of which may be isomorphous with the oxides of lead and strontian, the other with the oxide of iron.

" It must be confessed, however, that this is a subject upon which the immediate inferences from experiment seems to stand in direct opposition to all our preconceived opinions.

" At first sight, nothing would seem more obvious than that aggregates made up of the same number of atoms, agreeing in their primary properties, whether mechanical or chemical, shall produce the same substances; and this accordingly had been taken for granted in all discussions of the kind alluded to.

" Lately, however, various facts have come to light, which go to prove that bodies, which appear to possess precisely the same atomic constitution, may differ remarkably in their properties; nay, that they may even belong to a different class of substances altogether.

" Thus we have two distinct substances made up of carbon and hydrogen, in the proportion of 6 by weight of the former to 1 of the latter; the first a gaseous body, olefiant gas, the second a highly volatile liquid.

" Professor Berzelius, in a paper published in the Transactions of the Swedish Academy in 1830, has enumerated several other examples of the kind, distinguishing them by the name of *isomeric* bodies.

" The phosphoric acid is one of them; when exposed to a red heat for some time, it acquires new properties, coagulating albumen, and producing white instead of yellow precipitates with nitrate of silver.

" The tartaric acid is another case in point, Berzelius having discovered in certain kinds of tartar an acid differing in properties from, though agreeing in chemical constitution with, that more commonly known. The cyanous and fulminic acids are instances still more remarkable.

" Such are the principal examples of the kind taken from inorganic nature; but among organic bodies they would appear, from the researches of Dr. Prout, to be much more numerous.

" Thus the sugar from the cane, and from the urine of diabetic patients, agrees as nearly in point of composition with the sugar of milk, manna, and gum arabic, as the several varieties of cane-sugar do with each other; yet the first class of sugars yield oxalic, the second saclactic acid.

"Professor Stromeyer concludes, that this discrepancy arises from the dissimilar arrangement of the component atoms, and the different degrees of condensation they have undergone; but it appears to me more probable, that the presence of a portion of some principle, occasionally even too minute to be detected by analysis, may have occasioned the development of new properties. Dr. Prout is of opinion, that some foreign body, not of itself belonging to the animal or vegetable kingdoms, necessarily enters into the constitution of every substance capable of becoming assimilated, and constituting a part of any organic structure. Bodies containing this admixture he denominated *merorganized*, in order to express this supposed condition, implying that in passing into this state they become partly, or to a certain extent, organized.

"Now he accounts for the exceeding diversity of properties possessed by organic bodies, whose chemical composition is nearly identical, by the admixture of this small proportion of foreign matter, which by its presence infuses new properties into the mass, and prevents the particles from arranging themselves in their natural crystalline form.

"Thus starch is *merorganized* sugar, differing only from the latter by the presence of certain foreign matters, which effect a complete change in its characters.

"This curious view is rendered more intelligible by the important researches of Mr. Herschel, detailed in his Bakerian lecture for 1824; in which he has shown, that the relations of a mass of matter, such as mercury, to electricity, may be even reversed by the presence of an almost infinitesimal quantity of a substance, such as potassium, in an opposite electrical condition.

"'That such minute proportions of extraneous matter,' says Mr. Herschel, 'should be found capable of communicating sensible mechanical motions and properties of a definite character to the body they are mixed with, is perhaps one of the most extraordinary facts that has appeared in chemistry. When we see energies so intense exerted by the ordinary forms of matter, we may reasonably ask, what evidence we have for the imponderability of any of the powerful agents, to which so large a part of the activity of material bodies seems to belong.'" (P. 78.)

Upon this the author remarks:

"The views here promulgated promise to throw some light upon a subject in which I have long felt a lively interest, namely, the virtues of certain medicinal springs which contain but a very minute proportion of any solid ingredient. Much of their efficacy may doubtless be attributed to the influence of imagination, to change of scene and of habits, and in many cases to the transition from a dense and impure to a more clear and rarefied atmosphere. But, after all these deductions, there seems to be a residual phenomenon, to use a phrase of Mr. Herschel's, requiring a further explanation.

"Now it seems not improbable, that very minute portions of certain principles may act upon the system with an energy commensurate, not to their own quantity, but to the change their presence occasions in the properties of the more inert ingredients that accompany them.

"In this manner we may explain the powerfully tonic effects of certain springs containing a very minute impregnation of iron; the cures effected by waters, such as those of Loueche or Gastein, which appear to approach as nearly as possible to absolute purity; and the efficacy in glandular disorders attributed to certain others, in which a minute proportion of iodine or bromine has been detected.

"In a Memoir read before the Royal Society, on the saline and purgative springs of this country, in which I stated the proportions of iodine and bromine present in each, I expressed myself as being sceptical with regard to any medicinal agency that could be exerted by so small a quantity as one grain of iodine diffused through ten gallons of water, the largest quantity in which I had ever detected it. The considerations above stated now induce me to attach more importance to the circumstance of its presence, for it is just as possible, *à priori*, that this quantity of iodine should infuse new properties into the salts which accompany it, and cause them to act in a different manner upon the system, as that less than a millionth part of potassium should create so entire a change in the relations of a mass of mercury to electricity. Whether the waters of Cheltenham or Leamington affect the constitution differently from solutions of Glauber salt of similar strength, must be decided by the experience of those on the spot; but granting this to be the case, and there is not wanting testimony in favor of such an opinion, the discovery of these new principles in several of them may serve to explain their superiority.

"Dr. Prout has already hinted, in his Gulstonian lectures, at this solution, accounting in this manner for the fatal effects of inappreciable quantities of miasmata diffused through the atmosphere; nor is it unlikely that the system of the Homoiopathics in Germany may have grown out of some facts that had been observed, with respect to the powerful influence exerted on the system, when even very minute quantities of certain active principles were added to common medicaments." (P. 80.)

Who will say a word against infinitesimal doses after this?

In following out some of the applications of the theory of definite proportions, we observe that Dr. D. has brought some very curious circumstances together. Those respecting the classification of minerals are less striking than the botanical and astronomical examples; in the conclusion of the third chapter he says,

"It is possible, therefore, that at some future time a system of mineralogy, combining the advantages of a natural and an artificial method, may be proposed, which shall be based entirely on the atomic constitution of bodies, and the doctrine of isomorphism, which has proceeded from it, thus affording additional proof of the widely-spreading influence of this discovery; yet even at present we need not this further step to convince us, that the law of definite proportions extends throughout the whole of inorganic matter.

"Neither do vegetable or animal products appear exempt from its influence, although a provision exists, according to the ingenious views of Dr. Prout, for preventing its interference with the operations of life; a minute portion of some foreign matter being superadded to every definite compound intended to be assimilated, which, either by the interposition of its particles, or perhaps in some other less intelligible way, counteracts the operation of that cohesive attraction, the tendency of which is to impart somewhat of a crystalline character to the mass.

"It would, doubtless, be unphilosophical to attribute to the selfsame law of nature, the proportion existing between the particles that compose a compound body, and the relation of number that has been traced amongst the parts of the floral organs in plants; yet it may not be without interest to notice, as a proof of the analogy which runs throughout the whole of creation, and as indicating, perhaps, that the law of definite proportions itself, widely spreading as it seems, is but one of the consequences of some more comprehensive principle, the conclusions which the most distinguished botanists of the present day have arrived at, as to what ought to be regarded as the primitive types of monocotyledonous, dicotyledonous, and acotyledonous plants; these constituting the three great classes into which the vegetable kingdom may be divided.

"Mons. Decandolle observes, 'that the numbers 4, 5, and their multiples, appear to belong peculiarly to dicotyledonous plants; the number 3, and its multiples, to monocotyledonous; the number 2, and its multiples, to be established among the acotyledonous in the great family of mosses.' That exceptions from this standard are numerous will be evident, when we recollect that the main principle on which the artificial arrangement of Linnæus proceeds, is founded on the difference of number in the floral organs; but those who will take the trouble of perusing the philosophical remarks of the botanist alluded to on this subject, will, I flatter myself, rise with the conviction that these deviations from the supposed standard may be explained by the interference of other causes, such as the abortion of certain parts, or the adhesion of two or more, so as to have the appearance of one; whilst the existence of the same tendency towards regularity may be traced even in these, by an occasional return to the ideal structure, whenever the causes which usually interfere with it are accidently removed.

"Mons. Decandolle has also shown that this numerical proportion exists between the members of the different organs that together constitute the same flower, as well as between the component parts of different flowers belonging to the same class.

"Thus the relation of number between the parts of the calyx and corolla is very remarkable, and the deviations from this regularity there are met with may be referred to the causes above assigned, being most frequent where the parts are most numerous, the chances of abortion or of adhesion being increased in proportion.

"The same relation extends likewise to the stamens, subject to occasional deviations; and even in the pistillary system, which presents the greatest anomalies in this respect, it may be observed, that the number of the valves of the pericarp, of the placenta, of pistillary chord, of the styles, and the stigmas, &c., is always in the proportion of 1 to 1, 1 to 2, 2 to 1; so that one of these organs may serve to determine the rest, allowing for exceptions from abortion, &c.; and that, when the parts of the pistils are disposed in a whorl-shaped manner around an ideal or real axis, the number of their parts is in a determinate relation to that of the other parts of the flower, this relation being one of the following:

| | |
|---|---|
| 1 to 1. | 2—5 or its multiples. |
| 1—2 or its multiples. | 3—5 or ditto. |
| 1—3 or ditto. | 4—5 or ditto. |
| 1—5 or ditto. | 2—1. |
| 2—3 or ditto. | |

"Lastly, we learn from astronomers, that the members of the planetary system to which we belong, are themselves subject to a law of an analogous kind.

"Bode has observed, that the magnitudes of the several orbits which the planets describe bear a certain definite proportion one to the other, the distances of Mercury, Venus, the Earth, Mars, &c. from the sun being that of the numbers 4, 7, 10, 16, 28; so that the differences are as 3, 3, 6, 12. The law was interrupted between Mars and Jupiter, so as to induce him to consider a planet as wanting in that interval; a deficiency long afterwards supplied by the discovery of four new planets in that very interval, all of whose orbits conform in dimension to the law in question, within such moderate limits of error, as may be due to causes independent of those on which the law ultimately rests.*

"There cannot be a sublimer subject for contemplation, or one more calculated to elevate our ideas with respect to the Divine attributes, than the correspondence which may thus be traced between the laws that pervade the whole of creation, from the ultimate par-

* "In the third volume of the Cambridge Transactions, Mr. Challis has attempted to extend Bode's law of the distances of the planets from the sun, to the distances of the satellites from their respective primaries. He shows that the differences of the distances of Jupiter's satellites are very nearly in the ratio of $2\frac{1}{2}$; those of Uranus in that of $1\frac{1}{2}$; authorizing the conjecture that there are two undiscovered satellites between the 4th and 5th, and one between the 5th and 6th. In the case of Saturn's satellites, the ratio is further departed from, perhaps from the interference of the ring."

ticles of matter, which, by their extreme minuteness, baffle our very powers of conception, to those immense aggregates of them, which compose any one of the members of our own planetary system; and as, according to the grand conception of Boscovich, the attraction of gravitation, and that of cohesion, may perhaps turn out to be the same force exerted at different distances, so the various ways in which, as we have seen, the tendency to definite proportions (if I may so express myself) manifests itself throughout the whole of nature, will perhaps be eventually traced to the same law; of which what is called the atomic theory, comprehensive as it may be, is only one of the consequences.

"It is this, indeed, which constitutes the most striking distinction between the effects of art and of nature, the provisions of finite and of infinite intelligence; the former accomplishing its purposes by a multitude of particular contrivances and regulations, which being made to meet each circumstance as it arises, are inconsistent one with the other, and at the best are applicable to a limited number only, out of the infinite variety of possible cases; the other producing an immense series of effects by a few very simple laws, which not only harmonize exactly one with the other, but are afterwards found to be themselves the consequences of a still smaller number of first principles." (P. 98.)

The fourth chapter, which contains matter equally curious with that shown to exist in the three foregoing, we shall leave untouched, for we have already exceeded our purposed bounds; and the reader of the volume would blame us were we to anticipate all the good that it contains.

